# Supplementary material for: Dynamic transcriptomic profiles of zebrafish gills in response to zinc supplementation
Source: BMC Genomics. 2010 Oct 11;11:553. doi: 10.1186/1471-2164-11-553 (PMC3091702; doi:10.1186/1471-2164-11-553)
Supplement: Additional file 2 — Interactive Direct Interaction Network representing the molecular interactions between zinc, copper, iron, calcium and proteins encoded by transcripts changed by zinc supplementation. Mini web-site containing index.html and hyperlinked pages in subdirectory describing a Direct Interaction Network automatically generated based on curated interactions contained within the proprietary PathwayArchitect database. Ovals represent proteins and the circles symbolize metal ions. Objects are coloured by their abundance in zebrafish at the time-point they were significantly different from the control is a scale from -4 fold (dark green) to +4 fold (dark red). Where significant differences were found at more than one time-point, the colour overlay shows expression at the first instance. Dark blue squares denote 'binding', and light blue squares 'expression'; green squares stand for 'regulation', green diamonds for 'metabolism', and green circles for 'promoter binding'. Arrow heads indicate directionality of the interaction where annotated. All nodes and edges can be further interrogated by selecting the relative area of the image. [file 1471-2164-11-553-S2.zip › PathwayArchitect Zn xs DIN/463460.html]

# BINDING:

|  |  |
| --- | --- |
| Type | BINDING |
| Effect | None |


---

|  |  |
| --- | --- |
| Score | 0 |


---

|  |  |
| --- | --- |
| Reference Count | 11 |


---

|  |  |
| --- | --- |
| Mechanism | Unknown |


---

|  |  |
| --- | --- |
| Reference:0 || Sentence | "E1A interferes with the formation of this complex by occupying domains of Stat1 that bind to IRF1." |
| PMID | 10764778 |
| Year | 2000 |
| Species | Human |
| Journal | J Biol Chem |
| RefScore | 2 |
| Source | PArchNLP |
  |
|  |  |


---

|  |  |
| --- | --- |
 Reference:1 || Sentence | "Unphosphorylated Stat1 binds to IRF1 directly and we conclude that this complex uses the ICS-2/GAS element to mediate constitutive LMP2 transcription in vivo." |
| PMID | 10921891 |
| Year | 2000 |
| Species | Human |
| Journal | EMBO J |
| RefScore | 0 |
| Source | PArchNLP |
  ||  |  |


---

|  |  |
| --- | --- |
 Reference:2 || Sentence | "Conversely, IFN-gamma, but not IL-1 beta, induced signal transducer and activator of transcription (STAT) 1 and interferon regulatory factor (IRF)-1 binding." |
| PMID | 11742807 |
| Year | 2001 |
| Species | Rat |
| Journal | Am J Physiol Cell Physiol |
| RefScore | 1 |
| Source | PArchNLP |
  ||  |  |


---

|  |  |
| --- | --- |
 Reference:3 || Sentence | "STAT1 and STAT3 bind IRF-1 GAS, whereas STAT1, STAT2, IRF-1, and IRF-9 bind IRF-7 ISRE." |
| PMID | 14578356 |
| Year | 2003 |
| Species | Human |
| Journal | J Biol Chem |
| RefScore | 1 |
| Source | PArchNLP |
  ||  |  |


---

|  |  |
| --- | --- |
 Reference:4 || Sentence | "It was found that IFN-gamma-induced mechanisms, such as the binding of Stat1 to gamma activation sequences (GAS) and IFN regulatory factor-1 (IRF-1) to IFN-stimulated response elements (ISREs), are more highly activated following treatment with IFN and TNF-alpha." |
| PMID | 13678429 |
| Year | 2003 |
| Species | Human |
| Journal | J Interferon Cytokine Res |
| RefScore | 3 |
| Source | PArchNLP |
  ||  |  |


---

|  |  |
| --- | --- |
 Reference:5 || Sentence | IRF-2 constitutively binds to the two ISRE/IRF-E sites at the DRR, while IRF-1 and STAT1 are induced to bind to the two ISRE/IRF-E sites and the ISRE/IRF-E1, respectively, only after IFN-beta treatment. |
| Year | 2005 |
| PMID | 16301648 |
| Species | Mouse |
| Journal | J Immunol |
| RefScore | 2 |
| Source | PArchNLP |
  ||  |  |


---

|  |  |
| --- | --- |
 Reference:6 || Sentence | STAT1 and STAT3 bind IRF-1 GAS, whereas STAT1, STAT2, IRF-1, and IRF-9 bind IRF-7 ISRE. |
| Year | 2004 |
| PMID | 14578356 |
| Species | Human |
| Journal | J Biol Chem |
| RefScore | 1 |
| Source | PArchNLP |
  ||  |  |


---

|  |  |
| --- | --- |
 Reference:7 || Sentence | However, IFNbeta activation of IRF-1, which requires tyrosine-phosphorylated Stat1 homodimers binding to a gamma interferon activation sequence in its promoter is not affected by TSA. |
| PMID | 15194680 |
| Year | 2004 |
| Species | Human |
| Journal | J Biol Chem |
| RefScore | 1 |
| Source | PArchNLP |
  ||  |  |


---

|  |  |
| --- | --- |
 Reference:8 || Sentence | Nuclear concentrations of signal transducer and activator of transcription-1 (Stat1) and IFN regulatory factor-1 (IRF-1), transcription factors that bind gamma-activated sequences (GAS) and IFN-stimulated response elements (ISRE), respectively, were found to increase after stimulation with IFN-gamma and TNF-alpha relative to stimulation with individual cytokines. |
| PMID | 15684619 |
| Year | 2005 |
| Species | Human |
| Journal | J Interferon Cytokine Res |
| RefScore | 1 |
| Source | PArchNLP |
  ||  |  |


---

|  |  |
| --- | --- |
 Reference:9 || Sentence | Intracellular HIV-1 Tat protein represses constitutive LMP2 transcription increasing proteasome activity by interfering with the binding of IRF-1 to STAT1. |
| PMID | 16512786 |
| Year | 2006 |
| Species | Human |
| Journal | Biochem J |
| RefScore | 1 |
| Source | PArchNLP |
  ||  |  |


---

|  |  |
| --- | --- |
 Reference:10 || Sentence | In the present study, we investigated the molecular mechanism responsible for the Tat-induced LMP2 down-regulation and show that intracellular Tat represses transcription of the LMP2 gene by competing with STAT1 (signal transducer and activator of transcription 1) for binding to IRF-1 (interferon-regulatory factor-1) on the overlapping ICS-2 (interferon consensus sequence-2)-GAS (gamma-interferon-activated sequence) present in the LMP2 promoter. |
| PMID | 16512786 |
| Year | 2006 |
| Species | Human |
| Journal | Biochem J |
| RefScore | 3 |
| Source | PArchNLP |
  |


---

|  |  |
| --- | --- |
